# Paper-Based Microfluidic Device with a Gold Nanosensor to Detect Arsenic Contamination of Groundwater in Bangladesh

**DOI:** 10.3390/mi8030071

**Published:** 2017-03-01

**Authors:** Mosfera A. Chowdury, Noosheen Walji, Md. Almostasim Mahmud, Brendan D. MacDonald

**Affiliations:** Faculty of Engineering and Applied Science, University of Ontario Institute of Technology, 2000 Simcoe Street North, Oshawa, ON L1H 7K4, Canada; mosfera.chowdury@uoit.net (M.A.C.); noosheen.walji@uoit.ca (N.W.); md.mahmud@uoit.ca (M.A.M.)

**Keywords:** microfluidic paper-based analytical devices, arsenic, gold nanosensor, nanoparticles, Bangladesh, groundwater, drinking water

## Abstract

In this paper, we present a microfluidic paper-based analytical device (μPAD) with a gold nanosensor functionalized with α-lipoic acid and thioguanine (Au–TA–TG) to detect whether the arsenic level of groundwater from hand tubewells in Bangladesh is above or below the World Health Organization (WHO) guideline level of 10 μg/L. We analyzed the naturally occurring metals present in Bangladesh groundwater and assessed the interference with the gold nanosensor. A method was developed to prevent interference from alkaline metals found in Bangladesh groundwater (Ca, Mg, K and Na) by increasing the pH level on the μPADs to 12.1. Most of the heavy metals present in the groundwater (Ni, Mn, Cd, Pb, and Fe II) did not interfere with the μPAD arsenic tests; however, Fe III was found to interfere, which was also prevented by increasing the pH level on the μPADs to 12.1. The μPAD arsenic tests were tested with 24 groundwater samples collected from hand tubewells in three different districts in Bangladesh: Shirajganj, Manikganj, and Munshiganj, and the predictions for whether the arsenic levels were above or below the WHO guideline level agreed with the results obtained from laboratory testing. The μPAD arsenic test is the first paper-based test validated using Bangladesh groundwater samples and capable of detecting whether the arsenic level in groundwater is above or below the WHO guideline level of 10 μg/L, which is a step towards enabling the villagers who collect and consume the groundwater to test their own sources and make decisions about where to obtain the safest water.

## 1. Introduction

Bangladesh has an abundance of shallow and deep groundwater sources; however, due to the geology in the region, much of this water is contaminated with naturally occurring arsenic, which was first discovered in 1992 [1,2]. The World Health Organization (WHO) has described the arsenic contamination of groundwater in Bangladesh as “the largest mass poisoning of a population in history” [3]. In a recent study in 2010 [2], researchers analyzed 46,321 hand tubewells from 50 arsenic-contaminated districts out of a total of 64 districts in Bangladesh using laboratory testing (flow injection hydride generation atomic absorption spectrometry). They found that 48.1% of the wells had an arsenic level above the WHO guideline value of 10 µg/L and 30.9% of the wells were above the Bangladesh arsenic standard of 50 µg/L [2]. In 2001, it was estimated that 35 million people in Bangladesh were under potential risk of arsenic contamination, and over the following 10 years this number increased to approximately 80 million people [2,4]. A study performed in 2012 estimated that arsenic exposures to concentrations >50 μg/L and 10–50 μg/L account for 24,000 and perhaps as many as 19,000 adult deaths annually in Bangladesh, respectively [5]. The arsenic contamination problem is exacerbated by the temporal and depth variability of groundwater chemistry in Bangladesh, which varies seasonally and as a function of depth in the aquifers [6,7]. Arsenic contamination is a severe issue, which can regularly vary; therefore, it is critical for the users of the groundwater sources to have accurate information about the levels of arsenic in their hand tubewells.

In 1996, nation-wide testing was undertaken in Bangladesh to determine the arsenic levels for approximately 1 million hand tubewells, with trained personnel using field-test kits [8]. The field test kits used for this testing were based on the mercury bromide stain method, which was found to be incapable of producing reliable results for arsenic concentrations below 70 µg/L and had poor agreement with the results from lab-based testing methods [8]. Since this limit is higher than the Bangladesh arsenic standard of the time (50 µg/L), this may have resulted in the mislabeling of many of these hand tubewells as either unsafe red (>50 µg/L) or safe green (<50 µg/L) [8]. Other occurrences of arsenic testing of hand tubewells by local and international organizations in targeted regions of Bangladesh have used tests based on the mercury bromide stain method [2,8]. Field-test kits based on the mercury bromide stain method require multiple steps and have a side effect of producing toxic arsine gas while testing the water, and thus must be used by trained personnel. Therefore, there is a need for a test that can reliably test for arsenic levels at the WHO level of 10 µg/L, while also being convenient and safe to use, so that the users can regularly test their own hand tubewells. A test that is also inexpensive and disposable would further enable widespread and regular testing.

A number of researchers have implemented microfluidics technology to develop more reliable arsenic tests either by modifying existing methods or exploring new detection strategies. Successful examples include a PMMA-based compact disk biosensor using immobilized bacteria [9,10,11,12,13], electrophoretic techniques that rely on arsenic conjugation with selenium [14,15] and surface plasmon resonance techniques coupled with biotechnology [16]. Traditional microfluidic techniques have been successful in generating accurate and portable tests; however, paper-based platforms can offer less expensive, disposable, and user-friendly tests, which is especially important for tests targeted at individual users in developing countries.

Microfluidic paper-based analytical devices (µPADs) have received much attention recently and have high potential as reliable and inexpensive test platforms [17,18,19,20,21,22]. The µPADs can also be disposable, simple, portable, and easy to use with straightforward signal readouts for users [18]. Stocker et al. developed a paper-based biosensor for detecting arsenic in potable water, using bacterial biosensors based on a nonpathogenic laboratory strain of *Escherichia coli*, with a demonstrated detection limit of 8 µg/L [23]. Nath et al. developed a µPAD for detecting arsenic in drinking water using a gold nanosensor, which changes from red to a bluish-black color and is capable of detecting arsenic at 1 µg/L [24]. The µPADs with gold nanosensors are well-suited to arsenic tests aimed at the individual users due to their robustness, safety, and simple signal readout; however, the µPADs developed by Nath et al. were not tested using groundwater samples from Bangladesh, and when implementing similar chemistry into µPADs and testing with these groundwater samples, we found there to be interference from a number of naturally occurring metals in the groundwater samples, as described in this paper. Therefore, there is a need for a µPAD arsenic test that is capable of detecting whether the arsenic level in Bangladesh groundwater is above or below the WHO guideline level of 10 µg/L without interference from the naturally occurring metals.

**Figure 1:**
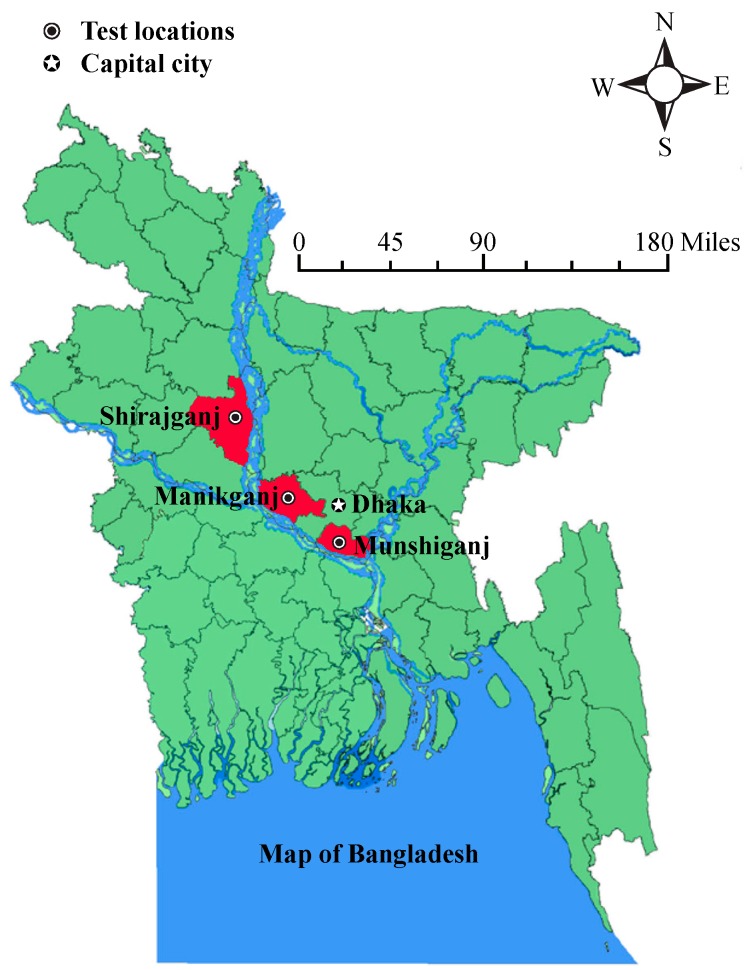
Map of Bangladesh with the three districts where groundwater samples were collected shown in red.

We have developed a µPAD that incorporates a gold nanosensor functionalized with α-lipoic acid and thioguanine (Au–TA–TG) to detect whether the arsenic levels of hand tubewells in Bangladesh are above or below the WHO arsenic guideline level of 10 µg/L. We present a successful strategy for preventing interference from the many naturally occurring metals in Bangladesh groundwater, including alkaline and heavy metals, which is based on adjusting the pH level. We collected 24 groundwater samples from three different districts in Bangladesh: Shirajganj, Manikganj, and Munshiganj, as shown in Figure 1. These samples were brought to our lab in Canada and tested with our µPADs, and the predictions of whether the arsenic levels in the samples were above or below the WHO guideline level of 10 µg/L were found to be in agreement with results from laboratory testing.

## 2. Materials and Methods

### 2.1. Materials

Gold (III) chloride trihydrate (HAuCl_4_·3H_2_O >99.9%), sodium citrate tribasic dihydrate (C_6_H_5_O_7_·2H_2_O·3Na >99.0%), (+)-α-lipoic acid synthetic (TA) (>99.0%, titration), EDC (*N*-(3-dimethylaminopropyl)-*N*′-ethylcarbodiimide hydrochloride, >99.0%), NHS (N-hydroxysuccinimide, 98%), 6-thioguanine (>98%) (TG), 0.25 M sodium hydroxide (NaOH), arsenic (As) standard for AAS (Atomic Absorption Spectroscopy) (As_2_O_3_), lead (Pb) standard for AAS, cadmium (Cd) standard for AAS, iron (Fe III) standard for AAS, ammonium iron (II) sulfate hexahydrate for preparing iron (Fe II) standard solution, nickel (Ni) standard for AAS, manganese (Mn) for AAS, sodium (Na) standard for AAS, magnesium (Mg) standard for AAS, calcium (Ca) standard for AAS, and potassium (K) standard for AAS were obtained from Sigma-Aldrich (Oakville, ON, Canada). Methanol was obtained from ACP Chemicals Inc. (Toronto, ON, Canada). The distilled water was obtained from Nothing But Water Products Inc. (Markham, ON, Canada). The cellulose chromatography paper (Whatman Grade 1 CHR by GE Healthcare, size: 20 cm × 20 cm, thickness: 0.18 mm) was obtained from Fisher Scientific (Philadelphia, PA, USA). Parafilm M™ Laboratory Wrapping Film (4 in. × 125 ft. roll) was obtained from Fisher Scientific, Mississauga, ON, Canada. Hydrochloric acid (HCl, 37%) and nitric acid (HNO_3_, 70%) were obtained from Sigma-Aldrich (Oakville, ON, Canada) and used for cleaning the glassware. Tap water used in the experiments was collected from our lab at the University of Ontario Institute of Technology (UOIT), between March and May 2016, in Oshawa, ON, Canada.

### 2.2. Gold Nanosensor Preparation

The protocol used to synthesize the gold nanoparticles was described by Steven D. Perrault, and is an adapted form of the Frens method reported by Walkey [25], with the steps detailed below. The gold nanosensor, with ligands, was prepared using a modified version of the protocol followed by Nath et al. [24]. Prior to the sensor preparation, all glass apparatuses were cleaned with aqua regia (HCl:HNO_3_ 3:1) and rinsed with distilled water at least 10 times. All chemical solutions were prepared with distilled water. For the gold nanoparticle (AuNP) synthesis, 2 mL of freshly prepared 25 mM HAuCl_4_·3H_2_O solution was added with 198 mL of distilled water. The mixture was stirred and boiled at 100 °C; afterwards, 2 mL of freshly prepared 3.3% (*w*/*v*) of C_6_H_5_O_7_·2H_2_O·3Na was added while stirring. The solution was stirred for an additional 15 min to ensure that the monodispersed gold nanoparticles remained a stable red color. To prepare the sensor, 45 mg of TA was dissolved into 10 mL of methanol and this solution was then added to 200 mL of freshly prepared gold nanoparticle solution at pH 8.0 (pH was adjusted using 0.25 M NaOH and measured with a ProLab 1000 pH meter) at room temperature. The solution was then stirred for 8 h. The Au–TA solution was then split into 10 mL volumes (for the centrifuge tubes) and centrifuged at 4000 rpm for 60 min (Eppendorf 5804). The excess solution was removed from each centrifuge tube with a pipette, leaving only the concentrated Au–TA at the bottom. The concentrated Au–TA collected from each centrifuge tube was dissolved with 5 mL of distilled water. A mixture of 2 mL of 10 mM EDC and 2 mL of 10 mM NHS was added with 100 mL of the diluted Au–TA at pH 8.0 (same pH adjustment as listed above) and stirred for 1 h. An amount of 90 mg TG was dissolved in 10 mL of 0.25 M NaOH and added to the previous solution after 1 h of stirring and left to stir for another 3 h. The final solution of conjugated Au–TA–TG was then centrifuged at 3500 rpm for 90 min (Eppendorf 5804). The excess solution was removed from each centrifuge tube with a pipette, leaving only the concentrated Au–TA–TG (the final gold nanosensor) at the bottom. The entire sensor preparation was performed inside a fume hood. As described by Nath et al. [24], the final gold nanosensor has free thiol (–SH) functional groups for arsenic recognition through As–S interaction.

### 2.3. Fabrication of μPAD and Test Method

**Figure 2:**
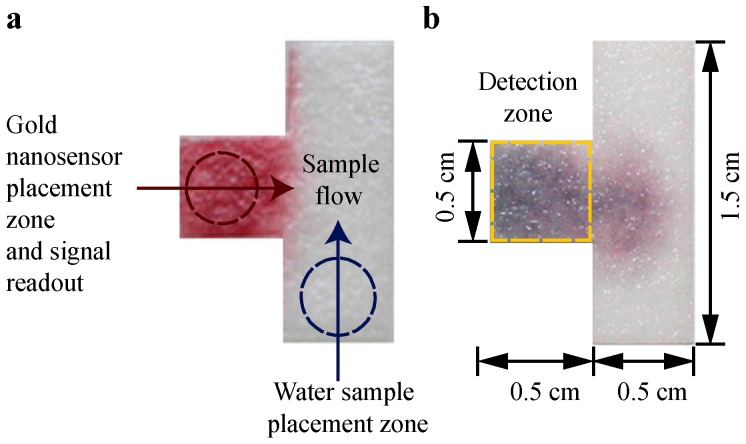
Images of T-shaped microfluidic paper-based analytical devices (µPADs), (**a**) with 0 µg/L arsenic, showing the red color and fluid placement zones; and (**b**) 50 µg/L arsenic showing the black color change, detection zone, and dimensions.

We used a T-shaped paper device, as shown in Figure 2, which was a sheet of Whatman CHR 1 cut by a CO_2_ laser engraver at 15 W (Speedy 100, 30 W, Trotec, Mississauga, ON, Canada). The T-shape was selected to provide a consistent and distinct detection zone while also allowing the sample to flow into the zone and interact with the gold nanosensor. Prior to testing, each paper strip was soaked in distilled water for 72 h in order to remove all impurities.

When an arsenic detection test was performed, the water sample was placed on the sample placement zone labelled in Figure 2a; then the gold nanosensor (Au–TA–TG) was placed on the gold nanosensor placement zone labelled in Figure 2a, both using a pipette. The specific volumes used for the gold nanosensor and further details for each test are provided in the Results and Discussion section. Immediately after the water sample and gold nanosensor were placed on the paper, within approximately 2 s, the µPAD was covered with a piece of Parafilm (4 cm × 4 cm) in order to prevent interference from the air, since the chemical sensor is sensitive to air. The µPAD was then scanned approximately 3.5 min after covering with the Parafilm to obtain the redness intensity. When the gold nanosensor interacts with arsenic, the red color will change to black due to the aggregation of the nanoparticles, as shown in Figure 2b.

### 2.4. Image Processing Using ImageJ

To analyze the test results, the µPADs were scanned in a desktop scanner (HP Deskjet 2540, Hewlett-Packard, Mississauga, ON, Canada) approximately 3.5 min after covering with Parafilm to generate a TIFF image. The scanned image was then opened in Image*J* and a box was drawn around the detection zone, which is designated by the yellow dashed rectangle shown in Figure 2b. Two mean RGB values were obtained from Image*J* for the detection zone area: the RGB value for the red pixels, and the mean RGB value (which is the average of the red, green, and blue pixel values), and is also called the gray pixel value. The red pixel value on its own is not enough information to assess the “redness” since the appearance of red coloring depends on the red value relative to the mean (gray) RGB value and not the absolute value. The mean (gray) value was subtracted from the red value to yield a value that corresponds to how much greater the red values is than the mean (gray) value, and thus indicates the “redness” of the area. This value is denoted as the “*relative red value*”. Since the gold nanosensor changes from red to black in the presence of arsenic, the relative red value was used as the metric to determine whether the concentration of arsenic in the water samples was above or below the WHO guideline level of 10 µg/L.

### 2.5. Groundwater Sample Collection and Analysis

For the testing of our µPADs, we collected water samples from hand tubewells in three districts in Bangladesh: Shirajganj, Manikganj, and Munshiganj, as shown in Figure 1. These three districts were shown to have arsenic contaminated hand tubewells, with approximately 33% of the wells in Shirajganj, 72% of the wells in Manikganj, and 93% of the wells in Munshiganj, found to contain arsenic above the WHO guideline level of 10 µg/L [2]. These three districts were selected to provide varying levels of contamination, as well as providing a variation of the metals naturally found in the aquifers.

We travelled to each of the districts, met with the local people who use the hand tubewells, and collected a total of 24 water samples from 24 hand tubewells by pumping the water into plastic bottles. Five samples were collected from Pathalia Para village in Shirajganj, nine samples from Bahirchar village in Manikganj, and ten samples from Mashakhola village in Munshiganj.

We brought 10 mL of each sample to Canada and separated each sample into 5 mL for the lab testing and 5 mL for the µPAD testing. The 5 mL groundwater samples were tested at a laboratory (York-Durham Regional Environmental Laboratory, Pickering, ON, Canada) using the well-established trace metal detection technique, Inductively Coupled Plasma Optical Emission Spectrometry (ICP-OES) method, to obtain the arsenic concentrations in each of the samples as a reference for the µPAD testing. The results of the laboratory testing are listed in Table 1.

## 3. Results and Discussion

### 3.1. Calibration of µPADs with Prepared Arsenic Solutions

The red to black color change was found to be sensitive to the volume of the gold nanosensor that was placed on the µPAD. We capitalized on this feature by using the volume to calibrate the µPAD to display a distinct red to black change at the WHO guideline level of 10 µg/L. We tested with six different volumes of gold nanosensor: 1.0, 1.2, 1.4, 1.6, 1.8 and 2.0 µL. Seven different concentrations of standard arsenic solution were used to perform the calibration: 1, 2, 5, 7, 10, 20 and 50 µg/L. A volume of 20 µL of water sample was used in this testing, since this value corresponded to the amount of fluid required to saturate the entire test strip.

**Figure 3:**
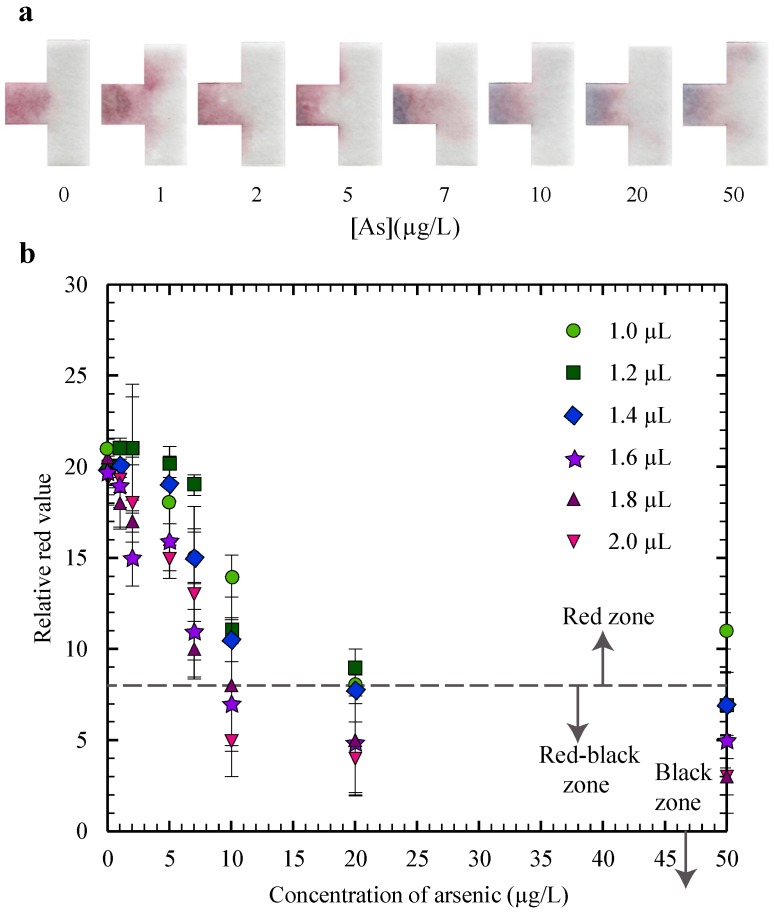
Results of the calibration tests showing, (**a**) the scanned strips after testing for the 1.6 µL volume; and (**b**) a plot of the relative red values for varying volumes of gold nanosensor.

**Figure 4:**
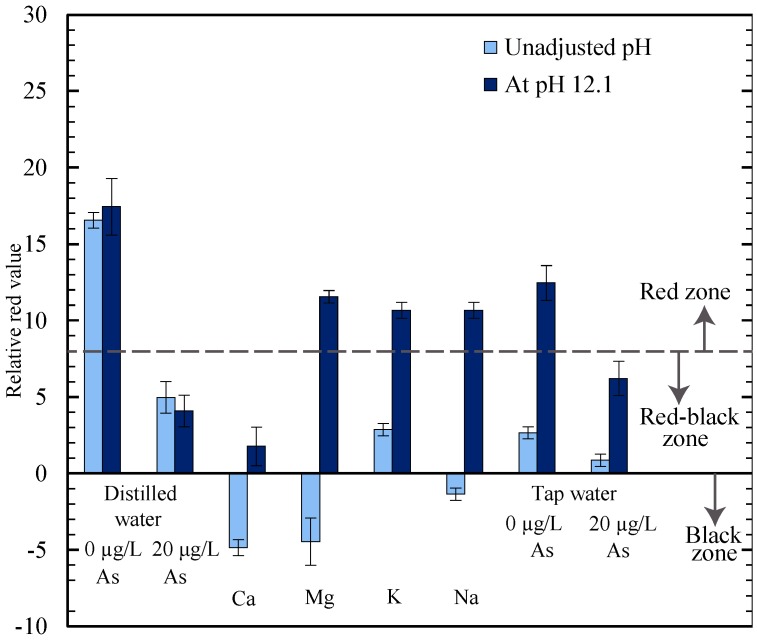
Interference testing with alkaline metal ions in distilled water solutions at groundwater concentrations (Ca at 28.50 mg/L, Mg at 11.22 mg/L, K at 8.10 mg/L, and Na at 10.10 mg/L) and combined together at Oshawa tap water concentrations (Ca at 37.50 mg/L, Mg at 9.90 mg/L, K at 1.70 mg/L, and Na at 13.00 mg/L).

**Figure 5:**
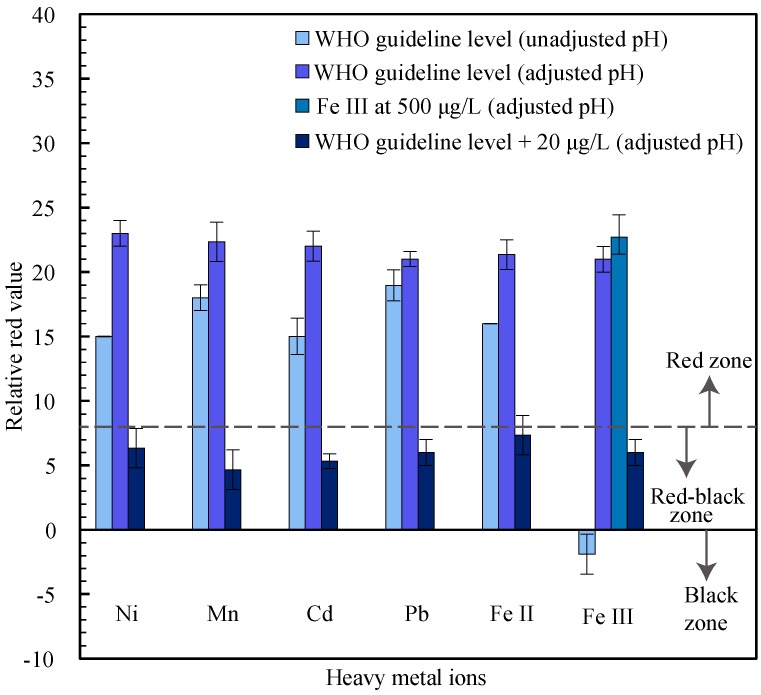
Interference testing with heavy metal ions at WHO guideline levels (Ni at 20 µg/L, Mn at 500 µg/L, Cd at 3.0 µg/L, Pb at 10 µg/L, Fe II at 300 µg/L and Fe III at 300 µg/L) with no arsenic (at unadjusted pH and with pH adjusted to 12.1) and with 20 µg/L arsenic (at adjusted pH), and a sample with Fe III at 500 µg/L (at adjusted pH).

**Figure 6:**
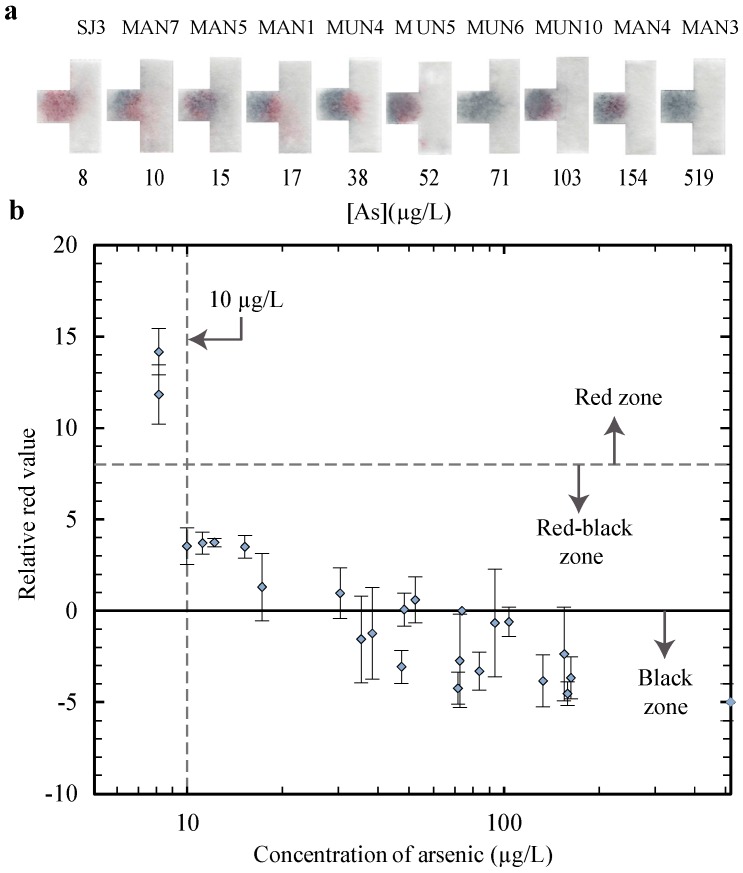
Results of the µPAD testing with groundwater samples collected in Bangladesh, showing (**a**) scanned images of some arsenic test strips to show representative color results; and (**b**) the relative red values resulting from the tests.

**Figure 7:**
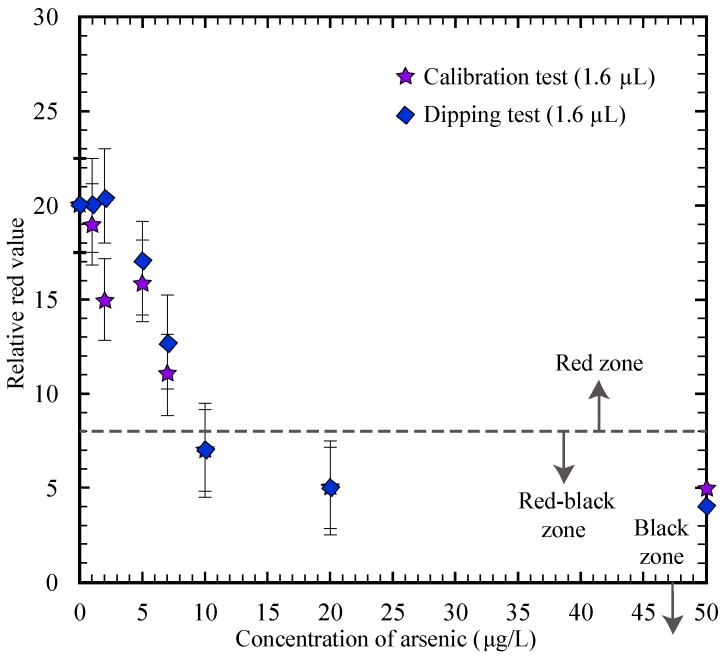
Comparison plot for the relative red values using 1.6 µL of gold nanosensor when the water sample is added by pipette (as in the calibration test) versus dipping the µPADs in the water sample until saturation.

The results plotted in Figure 3b were used to determine the volume of the sensor that most closely corresponded to a color change at 10 µg/L. To determine which relative red value corresponds to a visible shift from red to black, we correlated between the relative red values measured with the scanner and visual observation from the naked eye for the strips, with examples shown in Figure 3a. We had three different researchers examine a series of test strips with varying relative red values using naked eye determination, and determined that a noticeable shift from a red strip to a “red-black” strip corresponded to a relative red value of 8. The value of 8 was therefore selected as the reference value to indicate where a visible blackening of the strip occurs, and we denote values between 8 and 0 as the “red-black” zone. Since the “red-black” zone represents the visible signal for a shift from red to black, we can use this zone to denote a positive test result, indicating arsenic is present, and so throughout this study we use a relative red value of 8 as our demarcation point between positive and negative test results. A relative red value of 0 appears as having no red coloring, so we denote values of 0 and lower as the “black zone”. These zones are labelled in Figure 3b and a dashed line is shown at a relative red value of 8 to illustrate the division between the red zone and the red-black zone. For the plots throughout the manuscript, in Figure 3, Figure 4, Figure 5, Figure 6 and Figure 7, the data points correspond to the average value for three test repetitions, and the error bars correspond to one standard deviation (±σ).

Since a relative red value of 8 was selected as the visible demarcation point between positive and negative test results, we can select the volume of gold nanosensor from Figure 3b that results in an arsenic concentration below the WHO guideline level (10 µg/L) as having a relative red value above 8, and an arsenic concentration above the guideline level as having a relative red value below 8. From the results in Figure 3b, we see that both the 1.6 µL and 1.8 µL volumes corresponded most closely to the WHO guideline, with a relative red value just below 8 (inside the red-black zone) at a concentration of 10 µg/L. The 1.6 µL volume was selected since the results were similar and a smaller volume of gold nanosensor reduces the cost of the device. The relative red value continues to decrease for the 1.6 µL volume as the arsenic concentration is increased, indicating that the test is reliable at higher concentrations as well. We selected 1.6 µL as the volume of the gold nanosensor and used this value in all further testing performed in this study.

### 3.2. Interference Testing

#### 3.2.1. Alkaline Metals and pH Adjustment

The groundwater in Bangladesh has naturally occurring levels of alkaline metals such as Ca, Mg, Na and K. Our initial testing indicated that there was interference between these alkaline metals and the gold nanosensor. In order to analyze and eliminate the interference, we performed a series of experiments using lab-made solutions of each of the alkaline metals corresponding to the concentrations of these metals in our collected groundwater samples from the hand tubewells in Bangladesh. We used the ICP-OES method in the Chemistry Laboratory at UOIT to analyze the alkaline metal concentrations in one of our water samples, SJ3, and the results are summarized in Table 2. Later testing with the other water samples confirmed that they had similar interference behavior as sample SJ3, so we include the results for only this sample as a representative result. For comparison, we also tested the tap water from our lab, and listed the alkaline metal concentrations in Table 2.

In Figure 4, we plotted the results of the relative red value on the µPADs when the deposited water sample was a solution containing the concentrations listed in Table 2 for each of the individual alkaline metals. We also tested with both distilled water and tap water—one sample with no arsenic and one sample with 20 µg/L of arsenic added. A sample volume of 20 µL was used for the tests labelled as unadjusted pH. It can be seen in Figure 4 that all of the tested samples with alkaline metals present interfere with the gold nanosensor on the µPADs, since the results for the unadjusted samples were either in the red-black or black zone.

Domínguez-González et al. [26] described, how a change in the pH value due to the addition of samples with a different pH value than the gold nanosensor, can affect nanoparticle aggregation [26]. We decided to employ an adjustment in the pH value of the water samples as a strategy to eliminate the interference. We found that increasing the pH value of the sample to the same level as the gold nanosensor, a pH value of 12.1, counteracts the interference from the alkaline metals without altering the results for arsenic. We increased the pH on each µPAD to 12.1 by adding 2 µL 0.25 M NaOH solution on the paper strip in the “water sample placement zone” (labelled in Figure 2a) immediately before depositing each water sample. A 15 µL sample volume was used for the adjusted pH testing, which resulted in a total volume of 17 µL placed on the paper strip, including the volume of NaOH solution. This sample volume was selected for the adjusted pH testing since it resulted in the saturation of the entire test strip. There is a small dilution of the water sample due to the addition of 2 µL of NaOH solution, potentially reducing the concentration of metals in the water samples by a maximum of 12%. There was negligible impact from this dilution on the interference testing results or the predictions of whether the arsenic concentrations were above or below the WHO guideline levels. This dilution was within the error from the calibration testing, as shown in Figure 3, and was not significant enough to alter the test results.

The results for the distilled water samples indicate that the pH adjustment has a negligible influence on the relative red value when the metals are not present. The results of the pH adjusted tests in Figure 4 demonstrate that most of the alkaline metal solutions, Mg, K, and Na, had relative red values in the red zone, indicating that interference was significantly inhibited. For Ca, the pH adjustment for the pure solution of Ca was a substantial improvement, since the interference was reduced, but not enough that the result was in the red zone, as desired. By examining the tap water results in Figure 4, where a higher level of Ca was present, we see that the pH adjustment was successful in reducing the interference for this sample, and infer that the interaction of the ions together, along with other chemicals in the samples, prevents the Ca from interfering, and the result is that after pH adjustment there is insignificant interference from the alkaline metals. From examining the tap water sample where arsenic was added, the results indicate that the tests still responded to arsenic after the pH was adjusted, since the relative red value for this test was in the red-black zone as would be expected when 20 µg/L of arsenic is present.

#### 3.2.2. Heavy Metals

We also examined if there was interference from other heavy metal ions, which are commonly found in the groundwater of Bangladesh [4]. We tested the µPADs using sample solutions containing nickel (Ni), manganese (Mn), cadmium (Cd), lead (Pb), and iron (Fe II and Fe III), at WHO guideline levels (Ni: 20 µg/L, Mn: 500 µg/L, Cd: 3 µg/L, Pb: 10 µg/L, Fe II: 300 µg/L, and Fe III: 300 µg/L). Similar to the alkaline metal testing, a 20 µL sample volume was used for the unadjusted pH testing and a 15 µL sample volume was used for the adjusted pH testing. To analyze the interference of the heavy metals in the presence of arsenic, we tested each of these samples with no arsenic and with 20 µg/L of arsenic. When the groundwater samples were tested in a laboratory for arsenic concentrations using ICP-OES, as described in Section 2.5, we also obtained values for the concentration of Fe III and the other heavy metals (Ni, Mn, Cd, Pb, and Fe II). We found that some samples had concentrations of Fe III above the WHO guideline, with the highest concentration found in sample SJ8 at 495 µg/L. Therefore, we also ran an interference test for Fe III at a concentration of 500 µg/L, to assess the interference at relevant concentrations that can be encountered in Bangladesh groundwater. None of the other heavy metals (Ni, Mn, Cd, Pb, and Fe II) were found to have concentrations above the WHO guideline levels in the collected groundwater samples.

The results of the heavy metal tests are plotted in Figure 5, and we see that there is no interference for most of these heavy metals at the WHO guideline levels, with only the Fe III exhibiting interference. The tests also confirm that, except for Fe III, there is no inhibition of the arsenic signal due to the presence of the heavy metals, since the tests with 20 µg/L yielded relative red values at or below the red-black zone, as expected. Similar to the prevention of alkaline metal interference described in Section 3.2.1, we adjusted the pH value to 12.1 in order to prevent the interference from Fe III. From Figure 5, it can be seen that interference is successfully eliminated at the adjusted pH value for Fe III concentrations, both at the WHO guideline level, and the 500 µg/L level.

### 3.3. Testing Groundwater Samples with µPADs

We tested the 24 groundwater samples that were collected from the hand tubewells in Bangladesh using our µPADs. In order to incorporate our pH adjustment method, we deposited 2 µL of 0.25 M NaOH solution on the paper strip in the “water sample placement zone” (labelled in Figure 2a) immediately before depositing each water sample. A volume of 15 µL of water sample was used for these tests.

In Figure 6a, we show the scanned images of some tests to demonstrate how the tests above the WHO guideline level of 10 µg/L appear as “red-black” or black to the naked eye, and the tests below the WHO guideline level appear as red. Also, as the concentration increases from left to right, the µPADs appear progressively less red and more black. In Figure 6b, it can be seen that the µPADs yield a relative red value above 8 (in the red zone) for tests with an arsenic concentration below 10 µg/L, and the tests with a concentration above 10 µg/L have a relative red value below 8. These results confirm that our tests are capable of indicating whether actual groundwater samples collected from hand tubewells in Bangladesh are above or below 10 µg/L. This indicates that we have shown, for the first time, a paper-based test that can reliably predict arsenic contamination for groundwater in Bangladesh without interference from the naturally occurring metal ions.

### 3.4. Dipping the µPADs

The µPADs presented in this paper were tested using pipettes to measure the precise volumes of both the gold nanosensor and water sample. This method is not conducive to use by untrained users, so we analyzed a technique for more straightforward testing. Instead of using a pipette for the water sample, we ran the same calibration test described in Section 3.1 by dipping the end of each µPAD (the end near the water sample placement zone in Figure 2a) in a water sample until the water sample reached the far end of the paper strip, indicating saturation (this took approximately 10 s). The µPAD was removed from the water sample after the flow reached the end of the strip to avoid the possibility of significant post-wetting flow, as observed in a previous study [27]. A volume of 1.6 µL of the gold nanosensor was measured with a pipette, deposited on a plastic surface, then the paper strip was dipped into the gold nanosensor deposit (near the gold nanosensor placement zone in Figure 2a). The test results agreed with the previously obtained test results, as shown in Figure 7 (for 1.6 µL volume), thus validating this method. The dipping procedure provides a more user-friendly experience for testing with the µPADs.

### 3.5. Cost of the µPADs

Based on the pricing from our retail purchases, the cost of the chemicals required to prepare the 1.6 µL volume of gold nanosensor for each arsenic test is $0.0127 USD. Based on the retail price of paper, the cost of the T-shaped chromatography paper required for each test is $0.0035 USD. Therefore, the total cost of materials for each µPAD with a gold nanosensor, at retail pricing, is $0.0162 USD. For comparison, the cost of each test from a conventional retail field-test kit (e.g., Hach kit) can vary widely, but is approximately $1 USD per test (which includes additional costs such as packaging, distribution, and profit).

## 4. Future Work for Commercialization

Commercially viable tests would require further improvements on the µPADs presented in this manuscript. In this section, we discuss some of the recommended improvements and the associated future work required.

It would be advantageous for commercial arsenic tests to provide semi-quantitative results and naked eye detection has an inherent reduction in the precision of the test results. Since the results in Figure 6b show a trend towards continuously decreasing relative red values as the arsenic concentration increases, there may be potential to develop a test reading app on a mobile phone to provide semi-quantitative arsenic readings based on the µPAD tests, rather than relying on naked eye detection. Future work could involve the pairing of the µPAD and a mobile phone app to provide these semi-quantitative results onsite.

Dipping the tests in water samples removes the pipetting requirement for the water sample, but the gold nanosensor and NaOH still require precise measurement with a pipette. A future iteration of the test could enclose a precise volume of both the NaOH and gold nanosensor in two blister packs, thus also protecting them from air and light. A thin airtight plastic packaging could also enclose the µPAD to provide structural support and increase the robustness of the tests, while further preventing exposure to air. With respect to shelf-life, Nath et al. [24], reported that the gold nanosensor still works after 4 weeks of storage at 4 °C. Commercial viability of the µPAD arsenic test through further improvements will be crucial for widespread adoption.

## 5. Conclusions

We reported in this paper a T-shaped µPAD with a gold nanosensor functionalized with α-lipoic acid and thioguanine (Au–TA–TG) that successfully detected whether the arsenic level in the groundwater of Bangladesh was above or below the WHO guideline level of 10 µg/L. To the best of our knowledge, this was the first successful demonstration of a µPAD arsenic detection test with groundwater samples from Bangladesh. We employed a simple pH adjustment strategy to prevent interference from the naturally occurring alkaline metals. The pH of the water sample on the µPAD was increased to 12.1 during the testing to eliminate the alkaline metal interference. We found there to be no interference from most of the naturally occurring heavy metals, except for Fe III, which we were also able to prevent by increasing the pH level to 12.1. We visited three arsenic contaminated districts in Bangladesh: Shirajganj, Manikganj, and Munshiganj, and collected groundwater samples from hand tubewells for testing with our gold nanosensor µPAD. The µPAD predictions of whether the arsenic level was above or below the WHO guideline level of 10 µg/L correlated with results obtained from laboratory testing using ICP-OES. Our µPAD is a significant step towards providing arsenic contamination information to the users themselves and empowering them to make decisions about their water usage and reduce the arsenic contamination issue in Bangladesh.

## Figures and Tables

**Table 1:** Arsenic level of groundwater obtained by Inductively Coupled Plasma Optical Emission Spectrometry (ICP-OES) for three different districts of Bangladesh (Shirajganj, Munshiganj and Manikganj).

Location	Sample ID	Arsenic Concentration (µg/L)
Shirajganj	SJ3	8
	SJ2	11
	SJ5	12
	SJ4	30
	SJ1	35
Manikganj	MAN8	8
	MAN7	10
	MAN5	15
	MAN1	17
	MAN9	48
	MAN6	83
	MAN2	132
	MAN4	154
	MAN3	519
Munshiganj	MUN4	38
	MUN3	47
	MUN5	52
	MUN6	71
	MUN1	72
	MUN9	73
	MUN2	93
	MUN10	103
	MUN7	158
	MUN8	162

**Table 2:** Concentration of alkaline metal ions obtained from ICP-OES in Oshawa tap water and in groundwater collected from Shirajganj, Bangladesh.

Alkaline Metal Ions	Concentration in Oshawa Tap Water (mg/L)	Concentration in Shirajganj (SJ3) Groundwater (mg/L)
Ca	37.5	28.5
Mg	9.9	11.22
K	1.7	8.1
Na	13	10.1
